# *Origanum vulgare* Essential Oil against *Tenebrio molitor* (Coleoptera: Tenebrionidae): Composition, Insecticidal Activity, and Behavioral Response

**DOI:** 10.3390/plants10112513

**Published:** 2021-11-19

**Authors:** Angelica Plata-Rueda, José Cola Zanuncio, José Eduardo Serrão, Luis Carlos Martínez

**Affiliations:** 1 Department of Entomology, Federal University of Viçosa, Viçosa 36570-000, Brazil; angelicaplata@yahoo.com.mx (A.P.-R.); zanuncio@ufv.br (J.C.Z.); 2 Department of General Biology, Federal University of Viçosa, Viçosa 36570-000, Brazil; jeserrao@ufv.br

**Keywords:** gas chromatography, repellency, respiration rate, terpenoids, survivorship, yellow mealworm

## Abstract

*Tenebrio molitor* is one of the main stored product pests. This study characterized oregano essential oil (OEO) by gas chromatography (GC/FID and GC/MS) and assessed its insecticidal properties against *T. molitor*. Mortality, survival, respiration, and behavioral response in larva, pupa, and adult of this insect were determined. The major components of OEO were carvacrol (25.6%), p-cymene (12.3%), linalool (8.71%), thymol (7.22%), γ-terpinene (7.21%), caryophyllene oxide (4.67%), α-pinene (2.71%), and eucalyptol (2.69%). OEO caused high contact toxicity in larvae (LD_50_ = 3.03 µg insect^–1^), pupae (LD_50_ = 5.01 µg insect^–1^), and adults (LD_50_ = 5.12 µg insect^−1^) of *T. molitor*. Survival rates were 100% in larvae, pupae, and adults of *T. molitor* not treated with OEO, declining to 65–54%, 38–44%, 30–23%, and 6–2% in insects treated with LD_25_, LD_50_, LD_75_, and LD_90_, respectively. Low respiration rates of *T. molitor* at different developmental stages was observed after OEO exposure. Additionally, OEO exposure affects behavioral avoidance response and causes repellency in larvae and adults. These findings show that OEO exerts insecticidal and repellent effects against *T. molitor*, suggesting a potent alternative to synthetic insecticides for controlling the beetle.

## 1. Introduction

The yellow mealworm beetle, *Tenebrio molitor* (L.) (Coleoptera: Tenebrionidae) is one of the main stored product pests of grains, flour, bran, and pasta worldwide. *Tenebrio molitor* infests many stored agricultural commodities [1], contaminates the food with their feces and molted exoskeleton [2], and may act as vector of fungi [3], making them unfit for human consumption. Some methods to control *T. molitor* include temperature treatment [4], sun treatment [5], controlled atmosphere [6], and fumigation of chemical insecticides [7]. However, negative consequences such as development of insecticide resistance [8], environmental pollution [9], and residual toxicity [7] have limited the application of chemical control. The search for new strategies to pest control can be implemented to protection of stored products, considering the harmful effects of synthetic insecticides.

Plant essential oils (EOs) are proposed for pest control in stored grains and display several insecticidal activities [10]. EOs alter insect digestion, causing repellency, deterrence, and changes in olfactory responses [11,12,13]. On the other hand, effects on insect physiology involve inhibition of growth, developmental impairment, oxygen deprivation, and energy depletion [14,15,16]. EOs are a mixture of plant metabolites (monoterpenes, phenylpropenes, and sesquiterpenes) and act on the nervous system of insects, affect acetylcholine [17], γ-aminobutyric acid [18], and octopaminergic receptors [19], as well as respiratory pathways [20]. For instance, EOs can be applied on insects by contact (through the integumentary system) [14], inhalation (through the respiration system) [21], and orally (through the digestive system) [22].

The biological activity on insects caused by EOs varies according to plant species, and their efficacy has been demonstrated in coleopteran stored product pests [12,15,23]. Previous studies showed that *Cymbopogon citratus* essential oil is lethal to *Ulomoides dermestoides* Fairmaire (Tenebrionidae) [16], *Hazomalania voyronii* essential oil to *Tribolium confusum* (du Val) (Tenebrionidae) [24], and *Zingiber pupureum* essential oil to *Lasioderma serricone* Fabricius (Anobiidae) [25], favoring an effective approach toward Integrated Resistance Management (IRM). In this context, EOs from Amarydillaceae [26], Annonaceae [20], Lauraceae [27], Meliaceae [28], and Poaceae [29] are the most promising for causing toxic effects in insects.

Oregano, *Origanum vulgare* (L.) (Lamiales: Lamiaceae) is an important aromatic plant rich in terpenoid components used in medical treatments [30], the food industry [31], and agriculture [32]. Oregano essential oil (OEO) has been used for a long time as natural tool to protect against several microorganisms of stored products [33], with low animal toxicity and rapid degradation in environment. Among the antimicrobial properties, OEO exhibits potent insecticidal effects against stored pests [34]. In *T. molitor*, deleterious effects caused by synthetic insecticides have been investigated [35]; however, OEO might be used to manage *T. molitor* populations. We assume that OEO causes biocidal effects in larva, pupa, and adult of *T. molitor*, which could be due to its capability to affect the survival, respiration, and behavior of this pest.

The purpose of this research was to characterize the main components of OEO and assayed the mortality, survival, respiration, and behavioral effects on *T. molitor*. This contributed to understanding how this bio-insecticide controls the yellow mealworm and how it can serve to manage synthetic insecticide resistance.

## 2. Results

### 2.1. Chemical OEO Characterization

**Figure 1:**
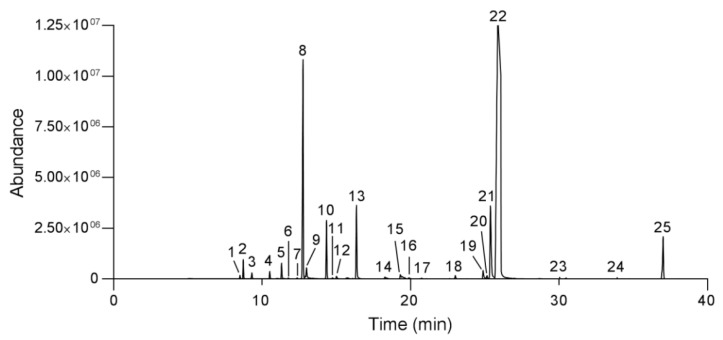
Gas chromatogram profiles of peak retention of components of oregano essential oil: α-thujene (1), α-pinene (2), camphene (3), β-pinene (4), β-myrcene (5), α-phellandrene (6), α-terpinene (7), p-cymene (8), eucalyptol (9), γ-terpinene (10), cis-sabinene hydrate (11), terpinolene (12), linalool (13), camphor (14), borneol (15), terpinen-4-ol (16), α-terpineol (17), thymol methyl ether (18), carvacrol methyl ether (19), cuminaldehyde (20), thymol (21), carvacrol (22), aromandrene (23), β-bisabolene (24), and caryophyllene oxide (25).

A total of 25 components were identified in OEO, accounting for 95.79% of the total composition: carvacrol (25.28 ± 0.34%), p-cymene (11.9 ± 0.33%), linalool (8.71 ± 0.66%), thymol (7.23 ± 0.22%), γ-terpinene (6.88 ± 0.38%), caryophyllene oxide (4.67 ± 0.08%), α-pinene (2.71 ± 0.02%), eucalyptol (2.66 ± 0.27%), camphene (1.96 ± 0.01%), α-phellandrene (1.87 ± 0.02%), β-pinene (1.71 ± 0.02%), thymol methyl ether (1.68 ± 0.18%), camphor (1.67 ± 0.09%), β-bisabolene (1.64 ± 0.08%), carvacrol methyl ether (1.64 ± 0.01%), terpinen-4-ol (1.56 ± 0.26%), borneol (1.46 ± 0.07%), α-thujene (1.42 ± 0.02%), terpinolene (1.37 ± 0.15%), aromandrene (1.35 ± 0.02%), cis-sabinene hydrate (1.35 ± 0.11%), α-terpineol (1.34 ± 0.19%), α-terpinene (1.23 ± 0.01%), cuminaldehyde (1.23 ± 0.01%), and β-myrcene (1.16 ± 0.02%) (Figure 1; Table 1).

### 2.2. Dose–Mortality Relationship

The dose–mortality data were suitable for a model probit fit (*p* > 0.05), demonstrating the toxicity of OEO to *T. molitor* and allowing estimates of toxicological endpoints (Table 2). The results indicated that OEO was the most toxic to *T. molitor* larvae (LD_50_ = 3.03 µg insect^−1^), followed by pupae (LD_50_ = 5.01 µg insect^−1^) and adults (LD_50_ = 6.12 µg insect^−1^). Mortality was less than 1% in the control.

### 2.3. Time–Mortality Relationship

**Figure 2:**
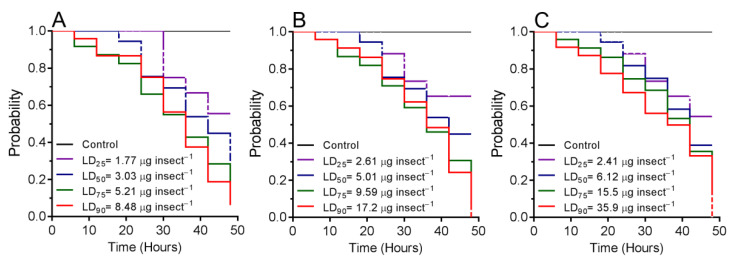
Survival curves of *Tenebrio molitor* exposed to different lethal doses of oregano essential oil, estimated using the Kaplan–Meier log-rank test. (**A**) Larva (*χ*^2^ = 20.22, *p* < 0.001), (**B**) pupa (*χ*^2^ = 18.71, *p* < 0.001), and (**C**) adult (*χ*^2^ = 15.31, *p* < 0.001).

Survival rates of *T. molitor* were calculated for 48 h after insect exposure to OEO at different lethal doses (Figure 2). Survival rates of *T. molitor* larvae differed significantly to OEO (log-rank test, *χ*^2^ = 20.22, df = 4; *p* < 0.001) and decreased from 99.9% (control) to 55.6% with LD_25_, 44.9% with LD_50_, 28.5% with LD_75_, and 6.31% with LD_90_. For *T. molitor* pupae, survival rate differed significantly (log-rank test, *χ*^2^ = 18.71, df = 4; *p* < 0.001) and decreased from 99.9% (control) to 65.3% with LD_25_, 53.9% with LD_50_, 30.7% with LD_75_, and 2.42% with LD_90_ of OEO. Survival rates of *T. molitor* adults differed significantly (log-rank test, *χ*^2^ = 15.31, df = 4; *p* < 0.001) and decreased from 99.9% (control) to 54.4% in insects exposed with LD_25_, 38.9% with LD_50_, 23.7% with LD_50_, and 3.32% with LD_90_ of OEO.

### 2.4. Respiration Rate

**Figure 3:**
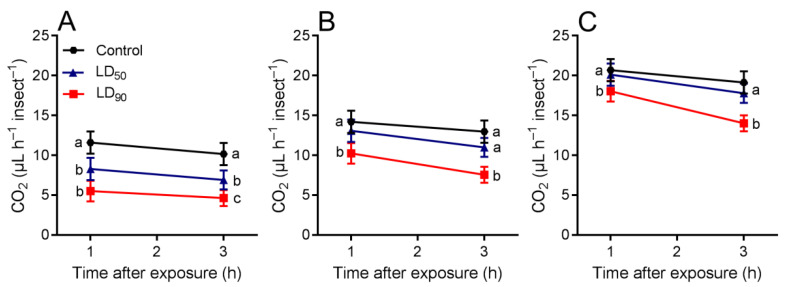
Respiration rate (mean ± SEM) of *Tenebrio molitor* in the control and after exposure to oregano essential oil at the LD_50_ and LD_90_. (**A**) Larva (**B**) pupa and (**C**) adult. Treatments means with different letters show significant differences by Tukey’s HSD test at the *p <* 0.05 level.

The respiration rate of *T. molitor* was influenced by exposure to OEO at the LD_50_ and LD_90_. Respiration rate of *T. molitor* larvae differed between the control group (11.6 μL CO_2_ h^−1^), LD_50_ (8.29 μL CO_2_ h^−1^), and LD_90_ (5.52 μL CO_2_ h^−1^) 1 h after exposure, but after 3 h, the respiration rate decreased to 10.1 μL CO_2_ h^−1^ in the control group, followed by LD_50_ with 6.91 μL CO_2_ h^−1^, and LD_90_ with 4.63 μL CO_2_ h^−1^ (Figure 3A). There was a significant effect of treatments (*p* < 0.001) and time (*p* < 0.001), but the interaction between treatments × time did not differ (*p* = 0.646) (Table 3). Respiration rate of *T. molitor* pupae differed between control group (14.2 μL CO_2_ h^−1^) and LD_50_ (13.1 μL CO_2_ h^−1^), and LD_90_ (10.2 μL CO_2_ h^−1^) 1 h after exposure. Respiration rates decreased from 12.9 μL CO_2_ h^−1^ in the control group to 10.9 μL CO_2_ h^−1^ in mealworm beetles exposed to LD_50_ and 7.55 μL CO_2_ h^−1^ with LD_90_ of OEO, after 3 h (Figure 3B). There was a significant effect of treatments (*p* < 0.001) and time (*p* < 0.001), but the interaction between treatments × time did not differ (*p* = 0.132) (Table 3). Respiration rates of *T. molitor* adult differed between the control group (20.6 μL CO_2_ h^−1^) and OEO with 20.1 μL CO_2_ h^−1^ to LD_50_ and 18.1 μL CO_2_ h^−1^ to LD_90_ 1 h after exposure. After 3 h, respiration rates decreased to 19.1 μL CO_2_ h^−1^ in the control group, 17.7 μL CO_2_ h^−1^ in mealworm beetles exposed to LD_50_ and 14.2 μL CO_2_ h^−1^ with LD_90_ of OEO (Figure 3C). There was a significant effect of treatments (*p* < 0.001), time (*p* < 0.001), but the interaction between treatments × time did not differ (*p* = 0.632) (Table 3).

### 2.5. Behavioral Avoidance Response

**Figure 4:**
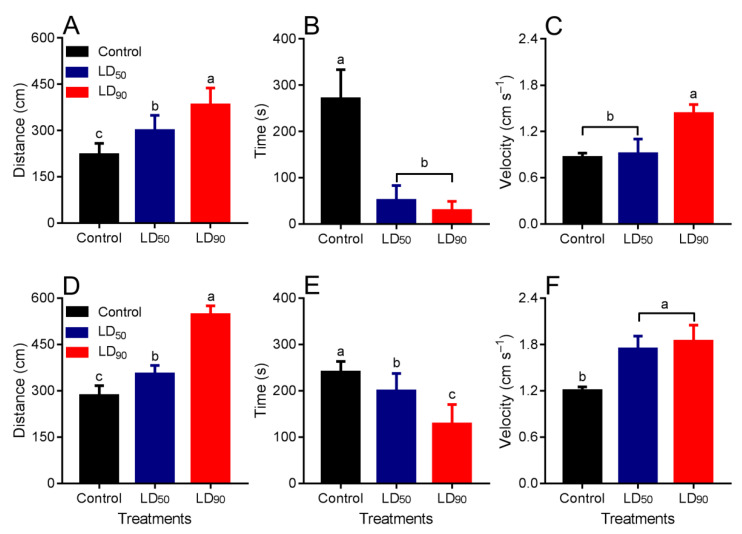
Behavioral response (mean ± SEM) of *Tenebrio molitor* exposed to oregano essential oil at different doses (control, LD_50_, and LD_90_) for 10 min. (**A**,**D**) Distance walked; (**B**,**E**) resting time; and (**C**,**F**) walking velocity. (**A**–**C**) larvae and (**D**–**F**) adults. Treatments means with different letters show significant differences by Tukey’s HSD test at the *p <* 0.05 level.

The distance traveled was different in the control and treated larvae (F_2,15_ = 51.05, *p* < 0.001) and *T. molitor* adults (F_2,15_ = 30.46, *p* < 0.001) with LD_50_ and LD_90_ of OEO (Figure 4A,D). The resting time was longer in the control than in the larvae exposed to LD_50_ and LD_90_ of OEO (F_2,15_ = 18.35; *p* < 0.001) (Figure 4B). Resting time differed between adults of the control, LD_50_ and LD_90_ of OEO (F_2,15_ = 26.01; *p* < 0.001) (Figure 4F). The walking velocity was lower in the control and LD_50_ larvae than in the LD_90_-treated ones (F_2,15_ = 5.88; *p* < 0.001) (Figure 4C). The walking velocity by adults were higher in the half-arenas treated with LD_50_ and LD_90_ of OEO (F_2,15_ = 9.21; *p* < 0.001) (Figure 4F).

## 3. Discussion

This work investigated OEO chemical composition and assessed the bioactive effects caused by this EO on *T. molitor* under laboratory conditions. Twenty-five components were identified. Carvacrol, p-cymene, linalool, thymol, γ-terpinene, caryophyllene oxide, α-pinene, and eucalyptol were the major components, in agreement with preliminary studies on terpenoids from OEO [36,37,38]. Specifically, majority OEO components are aromatic monoterpenes and can participate as kairomones or allomones in herbivore–plant chemical communication [39]. Terpenoids are metabolites with various biochemical mechanisms [40] and play a crucial role to induce defense responses against insects [41]. In particular, the insecticidal activity caused by majority OEO components was reported in insect pests such as *Drosophila melanogaster* Meigen (Diptera: Drosophilidae) in response to exposure to carvacrol [42], *Helicoverpa armigera* Hübner (Lepidoptera: Noctuidae) exposed to p-cymene and linalool [43,44], and *Sitophilus granarius* (L.) (Coleoptera: Curculionidae) exposed to thymol [45]. Regarding the mode of action, OEO presents little evidence of action of target proteins responsible for biological activity, but it is possible that its effect on the nervous system of *T. molitor* was due to the presence of terpenoids, resulting in rapid insect lethality, as reported for other insects exposed to EOs [27,29,46].

OEO caused mortality in *T. molitor* in a dose–dependent manner, as also studied in other insects exposed to EOs [47,48,49]. OEO was toxic to larvae (LD_50_ = 5.17 µg insect^−1^), pupae (LD_50_ = 5.17 µg insect^−1^), and adults (LD_50_ = 5.17 µg insect^−1^) of *T. molitor* and exerted a lethal effect by topical application. The different developmental stages of *T. molitor* exposed to high doses of OEO displayed altered locomotor activity, lost mobility, followed by paralysis and death. In this context, the symptoms of this insect were consistent with the recognizable effect on nervous system [19,35]. Exposure to OEO was shown to cause neurotoxicity in other insect pests as *Alphitobius diaperinus* (Panzer) (Coleoptera: Tenebrionidae) [50], *Nezara viridula* (L.) (Hemiptera: Pentatomidae) [51], and *Plutella xylostella* (L.) (Lepidoptera: Pyralidae) [52]. Overall, the results demonstrate that a low dose of OEO is sufficient to cause toxicity in *T. molitor* with potential as ecofriendly safe alternative to control this stored pest.

High variability in *T. molitor* survival is promoted by interaction of OEO attaching to the contact exposure and penetrating through respiratory system or insect cuticle, leading to the suppression nerve conduction. Short periods of exposure to OEO from 24 to 48 h, were needed to induce lethality in *T. molitor* and associated with the quick action of this bioinsecticide. In this research, the effects on *T. molitor* between lethal doses of OEO occurred at various periods. These time differences occur due to OEO components’ abilities to ingress the insect’s body during respiration [20] and penetrate the integument cuticle layers [15], exerting their effect as neurotoxins [17]. Similar effects were investigated in other pests of stored grains exposed to OEO, such as *Acanthoscelides obtectus* Say (Coleoptera: Chrysomelidae) [53], *Anagasta khueniella* Zeller (Lepidoptera: Pyralidae) [54] and *Rhyzopertha dominica* Fabricius (Coleoptera: Bostrichidae) [55]. The rapid effect against *T. molitor* is another indication of the potential of OEO to protect stored products.

OEO compromises the respiration of *T. molitor*, indicating physiological stress. Inhaled insecticides enter in the insect’s body through spiracles and tracheae and can affect the respiratory processes [13,16,21]. The regulated respiration occurs by the energy demand required to lead the detoxification enzymatic activity [12,47]. A decrease respiration results in high fitness cost and energy demand can be utilized to other metabolic functions [56]. A similar response occurs in other coleopteran pests such as *Demotispa neivai* Bondar (Coleoptera: Chrysomelidae) exposed to neem essential oil [10], *S. granarius* exposed to cinnamon essential oil [26], and *T. molitor* exposed to garlic essential oil [27] decreasing the oxygen consumption and disrupting of oxidative phosphorylation in respiration [13,21,57]. The findings obtained here, demonstrate that the larvae, pupae, and adults of *T. molitor* have low respiration rate when exposed to OEO with possible energy reallocated to other physiological functions and fitness costs.

The OEO also affected the behavioral response of *T. molitor*. Changes in the locomotion of *T. molitor* caused by OEO may be due to the toxic effect of this EO on the nervous system. Altered behavioral responses have been observed in different insects after toxic compounds exposure [22,29,58] with direct consequences on the orientation and olfactory response [59,60,61]. In *T. molitor*, larvae and adults exposed to contaminated surfaces by OEO gradually increase the distance walked, velocity, and reduce the resting time, indicating repellency. Indeed, the walking pattern changes in the different concentrations of OEO may be due to its feedback effect on neuron transmission through channels modulation that exerts action potentials along nerve axons and synapsis [35,62]. The findings show that changes in the locomotor activity of *T. molitor* are dependent on the concentrations of OEO, causing irritability.

## 4. Materials and Methods

### 4.1. Insects

*Tenebrio molitor* individuals were obtained from the Institute of Applied Biotechnology for Agriculture of the Federal University of Viçosa (Viçosa, Minas Gerais, Brazil). Larvae and adults were kept in plastic bottles (750 mL) at 26 ± 2 °C and 55 ± 25% relative humidity under a 12:12 h light/dark cycle. The insects were fed on wheat bran (*Triticum aestivum* Linnaeus, Poaceae), pieces of sugarcane (*Saccharum officinarum* Linnaeus, Poaceae), and chayote fruits (*Sechium edule* Jacquin, Cucurbitaceae). Newly-emerged last instar larvae, pupae, and adults (less than 24 h old) were employed in the experiments.

### 4.2. Essential Oil

OEO was obtained from *O. vulgare* (cv. *hirtum*) fresh plants and produced on an industrial scale by steam distillation (using a Clevenger-type apparatus) was supplied from Ferquina Industry and Commerce Ltd.a. (Catanduva, São Paulo, Brazil).

### 4.3. GC-FID and GC-MS Analysis

The OEO was analyzed in triplicate using gas chromatography (GC) to quantitative and qualitative chemical analyses. For quantitative analysis, GC analyses were conducted using a Shimadzu GC-17A Series instrument (Shimadzu Corporation, Kyoto, Japan) equipped with a capillary column (Supelco DB-5 30 m × 0.22 mm × 0.25 μm film) and Flame Ionization Detector (FID). The operation conditions were the following: carrier gas, helium at a flow rate of 1.5 mL min^−1^; injector temperature, 220 °C, detector temperature, 240 °C; column temperature to start at 40 °C (isothermal for 3 min), with a ramp of 3 °C min^−1^, to 240 °C, held isothermally at 240 °C for 12 min; injection, 0.1 mL (1% *w*/*v* in dichloromethane); split ratio, 1:10; and column pressure, 118 kPa. To each component identified, amount was determined as a relative percentage of total area of the chromatogram. For qualitative analysis, GC-MS analyses were performed on a Shimadzu GCMS-QP5050A chromatograph equipped with a Rtx-5MS capillary column (30 m, 0.25 mm × 0.25 mm; Restek Corporation, Bellefonte, PA, USA), coated with Crossbond (35% diphenyl and 65% dimethyl polysiloxane) and the same operative conditions used for GC-FID. A 0.1 mL aliquot of OEO (in 1% *w*/*v* in dichloromethane) was injected in splitless mode (1:10 ratio). Ionization was performed at 70 eV and mass spectral data were acquired in the scan mode in m/z range 40–400 Da. The OEO components were identified by comparison of their Kovats indexes from original literature [36,37,38], retention time and mass spectra data (with those of C_3_–C_24_ *n*-alkanes) obtained from National Institute of Standards and Technology (NIST08 and NIST11) libraries.

### 4.4. Dose—Mortality Relationship

The OEO was prepared in 2.5 mL of acetone to obtain six dilutions (1.56, 3.12, 6.25, 12.5, 25, and 50 µg insect^−1^). Serial dilutions and a control (acetone) were used to assess the toxicity of essential oil to *T. molitor*, construct dose–mortality curve and calculate lethal doses (LD_25_, LD_50_, LD_75_ and LD_90_). Acetone was employed as a control. For each OEO dilution, one microliter (1 µL) was applied on the body of one larva, pupa or adult of *T. molitor*, using a micropipette. Subsequently, the insects were individualized in Petri dishes (90 mm diameter), covered with a perforated cap for ventilation, and fed (larva or adult) on wheat bran. For each development stage, three replicates with 50 mealworm beetles were utilized for each OEO dilution and the number of dead insects was counted after 48 h of exposure.

### 4.5. Time—Mortality Relationship

The time–mortality for larvae, pupae, and adults of *T. molitor* using lethal doses (LD_25_, LD_50_, LD_75_, and LD_90_) obtained in the dose–mortality relationship of OEO was evaluated. *Tenebrio molitor* were exposed to the lethal doses and individualized in glass tubes (2.5 × 120 mm). Acetone was employed as a control. Three replicates of 50 mealworm beetles were utilized for each OEO lethal dose and the number of live insects was counted every 6 h for 2 d.

### 4.6. Respiration Rate

Respiration was evaluated for 3 h in individuals (larvae, pupae, and adults) of *T. molitor* after exposure to OEO (LD_50_ and LD_90_) according to the procedure of dose–mortality test and those untreated with acetone used as control. Carbon dioxide (CO_2_) (μL of CO_2_ h^−1^/insect) was measured with a respirometer of the type CO_2_ TR3C (Sable System Int., Las Vegas, NV, USA). One *T. molitor* (larva, pupa or adult) was introduced into a 25 mL glass chamber in a completely closed system. CO_2_ production was measured by 8 h at 26 ± 3 °C after insect acclimatization. Oxygen gas was injected through the glass chamber for 2 min at a flow of 150 mL min^−1^ to quantify the CO_2_ produced in the chamber. This airflow forces the CO_2_ molecules to pass through an infrared reader coupled to the system, allowing continuous measurement of the CO_2_ produced by insects in each chamber. Fifteen mealworm beetles of each developmental stage were used for LD_50_ and LD_90_ of OEO and control.

### 4.7. Behavioral Avoidance Response

Individuals (larvae and adults) of *T. molitor* were kept in Petri dish lined with filter paper (Whatman No. 1, Merck KGaA, Darmstadt, Germany) on the bottom, from here termed arena. To prevent the mealworm beetles escape, the inner part of the top of the Petri dish was coated with Teflon^®^ PTFE (E.I. Du Pont de Nemours & Co., Willmington, DE, USA). The behavioral avoidance response (distance walked, resting time, and walking velocity) was performed in arenas with half treated with 250 µL of OEO (LC_50_ and LC_90_) or control (acetone). The individual (larva or adult) *T. molitor* beetle was released in the center of an arena half-treated with OEO (in filter paper) for 10 min and recorded using the Videotrack automated system (ViewPoint Life Sciences, Montreal, QC, Canada). Sixteen *T. molitor* (larvae or adults) were used per OEO lethal dose or control. Mealworm beetles were considered irritated when they spent 50% of the time in half or repelled when they spent <1 min in half of the treated area with OEO [62,63].

### 4.8. Statistical Analysis

The dose–mortality relationship was determined with probit analysis using with SAS v.9.0 software [64] to estimate the lethal dose values with 95% confidence intervals. Time-mortality relationship was submitted to Kaplan–Meier survival analysis with GraphPad Prism v.7.1 software [65]. Respiration rate data were submitted to two-way analysis of variance (ANOVA) followed by Tukey’s HSD (Honestly Significant Difference) test (*p* < 0.05). The behavioral avoidance response of *T. molitor* was subjected to one-way ANOVA and means compared with the Tukey’s test (*p* < 0.05). Data on respiration rate and behavioral avoidance response were analyzed using with SAS 9.0 software. Raw data are availability in supplementary material (Table S1).

## 5. Conclusions

Overall, the results indicate that OEO has a wide range of detrimental effects on *T. molitor*. OEO caused toxicity, low survival, reduced respiration rate, and altered behavioral responses in different developmental stages. The chemical characterization shows that terpenoids contained in OEO may act synergistically through integument or respiratory systems to exert neurotoxicity on *T. molitor*. Furthermore, this research suggests that OEO is not only an alternating source of synthetic insecticides, but also might be utilized as innovative tool for effectively managing *T. molitor* populations.

## Figures and Tables

**Table 1:** Chemical composition of oregano essential oil. Ri, retention indices; Rt, retention time; MC, mean composition (% area); MM, molecular mass; *m*/*z*, mass/charge ratio; ID, identification methods; KI, Kovats (Ri^1^ = relative to standard mixture of *n*-alkanes in the same sample’s analytical conditions, Ri^2^ = from literature) retention indexes on a DB-5 column and compared with the literature [36,37,38]; MS, mass spectra.

Peaks	Components	Rt	MC	MM	*m*/*z*	KI		ID
						Ri^1^	Ri^2^	
1	α-thujene	8.40	1.42	152	93.05	930	930	KI [36,37,38], MS
2	α-pinene	8.56	2.71	136	93.05	938	939	KI [36,37,38], MS
3	Camphene	9.12	1.96	136	94.05	948	954	KI [36,37,38], MS
4	β-pinene	10.3	1.71	136	93.05	977	979	KI [36,37,38], MS
5	β-myrcene	11.1	1.16	136	94.05	987	991	KI [36,37,38], MS
6	α-phellandrene	11.5	1.87	136	98.95	997	1003	KI [36,37,38], MS
7	α-terpinene	12.1	1.23	136	98.95	1017	1018	KI [36,37,38], MS
8	p-cymene	12.4	12.3	134	119.0	1027	1025	KI [36,37,38], MS
9	Eucalyptol	12.8	2.69	154	108.1	1029	1031	KI [36,37,38], MS
10	γ-terpinene	14.1	7.21	136	115.0	1065	1066	KI [36,37,38], MS
11	Cis-sabinene hydrate	14.6	1.35	138	94.05	1078	1089	KI [36,37,38], MS
12	Terpinolene	15.8	1.37	136	95.05	1084	1089	KI [36,37,38], MS
13	Linalool	16.1	8.71	154	91.05	1098	1100	KI [36,37,38], MS
14	Camphor	18.1	1.67	152	95.05	1149	1146	KI [36,37,38], MS
15	Borneol	19.2	1.46	154	95.05	1175	1169	KI [36,37,38], MS
16	Terpinen-4-ol	19.6	1.56	154	91.05	1179	1177	KI [36,37,38], MS
17	α-terpineol	20.5	1.34	154	59.05	1192	1189	KI [36,37,38], MS
18	Thymol methyl ether	22.7	1.68	164	149.1	1238	1235	KI [36,37,38], MS
19	Carvacrol methyl ether	24.7	1.64	164	148.1	1248	1245	KI [36,37,38], MS
20	Cuminaldehyde	25.0	1.23	148	135.0	1247	1242	KI [36,37,38], MS
21	Thymol	25.1	7.22	150	135.0	1287	1290	KI [36,37,38], MS
22	Carvacrol	25.5	25.6	150	135.0	1305	1299	KI [36,37,38], MS
23	Aromandrene	30.2	1.35	204	135.0	1445	1441	KI [36,37,38], MS
24	β-bisabolene	34.1	1.64	204	99.05	1508	1506	KI [36,37,38], MS
25	Caryophyllene oxide	36.6	4.67	220	93.05	1581	1583	KI [36,37,38], MS

**Table 2:** Lethal doses of oregano essential oil on different developmental stages of *Tenebrio molitor* after 48 h exposure, obtained from probit analysis (df = 5). The chi-square value refers to the goodness of fit test at *p* > 0.05.

Insect Stage	No. Insects	Lethal Dose	Estimated Dose (µg Insect^–1^)	95% Confidence Interval (µg Insect^–1^)	Slope ± SE	χ^2^ (*p*-Value)
Larva	150	LD_25_	1.770	1.404–2.120	2.875 ± 0.29	2.23 (0.69)
	150	LD_50_	3.039	2.578–3.558		
	150	LD_75_	5.216	4.413–6.402		
	150	LD_90_	8.482	6.849–11.35		
Pupa	150	LD_25_	2.614	2.055–3.172	2.389 ± 0.22	3.38 (0.49)
	150	LD_50_	5.018	4.199–5.965		
	150	LD_75_	9.593	7.927–12.14		
	150	LD_90_	17.22	13.43–24.05		
Adult	150	LD_25_	2.412	1.726–3.114	1.166 ± 0.17	5.81 (0.21)
	150	LD_50_	6.124	4.895–7.713		
	150	LD_75_	15.54	11.86–22.34		
	150	LD_90_	35.96	24.62–62.26		

**Table 3:** Two-way ANOVA for respiration rate of *Tenebrio molitor* upon exposure to lethal doses (LD_50_ and LD_90_) of oregano essential oil at two times. DF = Degrees of freedom, SS = Sum of squares, MS = Mean square, *n* = numerator, d = denominator, *p* = probability of significance (*p* < 0.05).

Insect Stage	ANOVA Table	SS	DF	MS	F (DFn, DFd)	*p*-Value
Larva	Treatments	338	2	169	F (2, 54) = 154.1	<0.001
	Time	23.1	1	23.1	F (1, 54) = 21.12	<0.001
	Treatments × time	0.95	2	0.47	F (2, 54) = 0.44	=0.646
	Residual	59.1	54	1.09		
	Total	421	59			
Pupa	Treatments	228	2	114	F (2, 54) = 89.79	<0.001
	Time	60.4	1	60.4	F (1, 54) = 47.51	<0.001
	Treatments × time	5.34	2	2.67	F (2, 54) = 2.11	=0.132
	Residual	68.7	54	1.27		
	Total	363	59			
Adult	Treatments	163	2	81.8	F (2, 54) = 6.75	<0.001
	Time	103	1	103	F (1, 54) = 5.99	<0.017
	Treatments × time	15.9	2	7.95	F (2, 54) = 0.46	=0.632
	Residual	930	54	17.2		
	Total	1213	59			

## Data Availability

Not applicable.

## Supplementary Materials

The following are available online at https://www.mdpi.com/article/10.3390/plants10112513/s1, Table S1: Raw data. Data information of each experiment was supplied regarding data availability.
