# Supplementary material for: Origanum vulgare Essential Oil against Tenebrio molitor (Coleoptera: Tenebrionidae): Composition, Insecticidal Activity, and Behavioral Response
Source: Plants (Basel). 2021 Nov 19;10(11):2513. doi: 10.3390/plants10112513 (PMC8622527; doi:10.3390/plants10112513)
Supplement: Supplementary file 1 [file plants-10-02513-s001.zip › plants-1456520-supplementary.pdf]

Supplementary material
Table S1: Raw data

Experiment 1. Dose-response relationship

Dose	No. insects	Larva	Pupa	Adult
0	150	0	0	0
1.56	150	12	9	18
3.12	150	48	27	36
6.25	150	96	54	54
12.5	150	126	108	72
25	150	150	126	108
60	150	150	150	144

Experiment 2. Time-mortality relationship

Larva

Time (Hours)	Control	Time (Hours)	LD25= 1.77 mg insect-1	Time (Hours)	LD50= 3.03 mg insect-1	Time (Hours)	LD75= 5.21 mg insect-1	Time (Hours)	LD90= 8.48 mg insect-1
0	0	0	0	0	0	0	0	0	0
6	0	6	0	6	0	6	1	6	1
6	0	6	0	6	0	6	1	6	0
6	0	6	0	6	0	6	0	6	0
12	0	12	0	12	0	12	0	12	1
12	0	12	0	12	0	12	1	12	0
12	0	12	0	12	0	12	0	12	1
18	0	18	0	18	0	18	0	18	0
18	0	18	0	18	1	18	0	18	0
18	0	18	0	18	0	18	1	18	0
24	0	24	0	24	1	24	1	24	1
24	0	24	0	24	1	24	1	24	1
24	0	24	0	24	1	24	1	24	0
30	0	30	1	30	1	30	1	30	1
30	0	30	1	30	0	30	0	30	1
30	0	30	1	30	0	30	1	30	1
36	0	36	1	36	0	36	0	36	1
36	0	36	0	36	1	36	1	36	1
36	0	36	0	36	1	36	1	36	1
42	0	42	0	42	0	42	0	42	1
42	0	42	1	42	1	42	1	42	1
42	0	42	0	42	0	42	1	42	1
48	0	48	0	48	1	48	1	48	0
48	0	48	0	48	0	48	1	48	1
48	0	48	0	48	0	48	0	48	1

Pupa

Time (Hours)	Contr ol	Time (Hours)	LD25= 2.61 mg insect-1	Time (Hours)	LD50= 5.01 mg insect-1	Time (Hours)	LD75= 9.59 mg insect-1	Time (Hours)	LD90= 17.2 mg insect-1
0	0	0	0	0	0	0	0	0	0
6	0	6	0	6	0	6	0	6	1
6	0	6	0	6	0	6	1	6	0
6	0	6	0	6	0	6	0	6	0
12	0	12	0	12	0	12	0	12	1
12	0	12	0	12	0	12	1	12	0
12	0	12	0	12	0	12	1	12	0
18	0	18	1	18	0	18	0	18	0
18	0	18	0	18	1	18	0	18	1
18	0	18	0	18	0	18	1	18	0
24	0	24	1	24	1	24	1	24	1
24	0	24	0	24	1	24	0	24	1
24	0	24	0	24	1	24	1	24	0
30	0	30	1	30	1	30	1	30	1
30	0	30	0	30	0	30	0	30	0
30	0	30	1	30	0	30	1	30	1
36	0	36	0	36	0	36	0	36	1
36	0	36	1	36	1	36	1	36	1
36	0	36	0	36	1	36	1	36	0
42	0	42	0	42	0	42	0	42	1
42	0	42	0	42	1	42	1	42	1
42	0	42	0	42	0	42	1	42	1
48	0	48	0	48	0	48	1	48	1
48	0	48	0	48	0	48	1	48	1
48	0	48	0	48	0	48	0	48	1

Adult

Time (Hours)	Contr ol	Time (Hours)	LD25= 2.61 mg insect-1	Time (Hours)	LD50= 5.01 mg insect-1	Time (Hours)	LD75= 9.59 mg insect-1	Time (Hours)	LD90= 17.2 mg insect-1
0	0	0	0	0	0	0	0	0	0
6	0	6	0	6	0	6	0	6	1
6	0	6	0	6	0	6	1	6	0
6	0	6	0	6	0	6	0	6	1
12	0	12	0	12	0	12	0	12	1
12	0	12	0	12	0	12	1	12	0
12	0	12	0	12	0	12	0	12	0
18	0	18	1	18	0	18	0	18	1
18	0	18	0	18	1	18	0	18	1
18	0	18	0	18	0	18	1	18	0
24	0	24	1	24	1	24	1	24	1
24	0	24	0	24	0	24	0	24	1

24	0	24	0	24	1	24	1	24	0
30	0	30	1	30	1	30	0	30	1
30	0	30	0	30	0	30	0	30	0
30	0	30	1	30	0	30	1	30	1
36	0	36	0	36	0	36	0	36	0
36	0	36	1	36	1	36	1	36	1
36	0	36	0	36	1	36	1	36	0
42	0	42	0	42	0	42	0	42	0
42	0	42	1	42	1	42	1	42	1
42	0	42	0	42	1	42	1	42	1
48	0	48	0	48	0	48	0	48	1
48	0	48	0	48	0	48	1	48	1
48	0	48	0	48	0	48	0	48	1

Experiment 3. Respiration rate

		Time	1	2			Time	1	2			Time	1	2
		1	10.93499	10.7203			1	13.66817	12.75116			1	16.76968	12.44762
		2	11.65901	10.41823			2	12.1487	11.22211			2	23.24817	12.11786
		3	12.99605	9.921778			3	13.86393	12.77051			3	22.17932	16.13709
		4	12.72641	11.70785			4	13.9932	12.35241			4	23.29368	40.388
	Control	5	11.56222	10.08194		Control	5	13.3423	12.71475		Control	5	17.12949	15.62743
		6	10.72369	9.582174			6	13.67985	12.55413			6	23.24817	22.11786
		7	11.96486	10.4163			7	15.95317	13.95303			7	22.17932	21.13709
		8	10.07715	9.049567			8	16.38126	15.08759			8	18.29368	15.388
		9	10.72369	9.582174			9	14.12882	12.92571			9	17.12949	15.62743
Larva		10	12.65424	10.07938	Pupa		10	14.87882	13.39577	Adult		10	23.29368	20.388
		1	9.033552	6.566706			1	12.58427	8.745387			1	17.86737	16.58476
		2	7.141409	5.40112			2	11.65592	10.29434			2	21.26543	19.82936
		3	5.997087	3.97808			3	13.8437	11.05058			3	15.35273	13.62493
		4	7.978933	6.817453			4	13.26475	9.833053			4	23.06597	20.23583
	LD50	5	8.985889	7.749089		LD50	5	13.11583	12.34802		LD50	5	19.25355	16.62393
		6	9.957933	9.118033			6	13.03703	9.53633			6	23.95367	22.00735
		7	7.141409	5.40112			7	13.60626	12.67686			7	21.8554	22.23967
		8	8.985889	7.749089			8	12.46079	11.61942			8	21.43438	19.57032
		9	9.957933	9.118033			9	12.94607	10.763			9	17.66755	12.76333
		10	7.745417	7.100277			10	14.32107	12.888			10	19.3482	14.3482
		1	5.823067	5.10997			1	10.15618	9.285595			1	19.71705	13.67635
		2	5.796144	5.11961			2	11.39505	9.2904			2	17.00313	15.10894
		3	4.609667	3.629132			3	9.549889	6.986499			3	16.78693	14.5629
		4	4.573733	3.774144			4	9.999966	6.067473			4	19.93485	18.74285
	LD90	5	5.794833	4.748593		LD90	5	10.21482	7.542058		LD90	5	18.13547	12.64297

		6	5.5329	5.08252			6	12.23908	6.84803			6	18.77657	12.46413
		7	5.796144	5.11961			7	9.200382	8.507185			7	17.95173	14.85157
		8	5.794833	4.748593			8	10.83221	6.812687			8	16.46793	10.53803
		9	5.5329	5.08252			9	10.44845	7.667491			9	16.55983	10.97542
		10	5.997087	3.97808			10	8.335946	6.529991			10	18.92193	16.62393

Experiment 4: Behavioral avoidance response

Larva

Resting time			Walked distance			Walking velocity		
Control	LD50	LD90	Control	LD50	LD90	Control	LD50	LD90
231	20.4	52.4	211.8	283.1	303.1	0.916883	1.387745	2.578435
258	73.9	10.7	214.7	243.2	321.8	0.832171	0.329093	3.007477
346.7	18.7	51.2	267.7	308.4	392.2	0.772137	1.649198	2.766016
265.4	32.4	5.6	198.6	332	380.6	0.748304	1.024691	2.796429
245.5	50.2	25.8	205.3	302.5	356.1	0.836253	0.60259	2.380233
328.4	62.4	52.4	245.3	335.3	392.1	0.746955	0.53734	2.748282
363	32.9	10.7	225.9	258.9	316.3	0.622314	0.78693	2.956075
277.3	135.8	51.2	298.1	299.9	418.4	1.075009	0.220839	2.817188
316	51.9	5.6	261.4	208.1	433.8	0.827215	0.400963	3.746429
140.1	72	25.8	235.3	394.7	472.4	1.679515	0.548194	1.831008
366.1	50.1	30.8	231.7	340.8	369.7	0.632887	0.68024	2.200325
230.9	12.2	52.4	245.1	228	358.2	1.061498	1.868852	2.683588
283.6	74.9	10.7	173.9	321.2	308.4	0.613188	0.428838	2.882243
198.9	50.2	51.2	173.7	295.8	374.4	0.873303	0.589243	2.73125
210.8	73.8	5.6	171.3	366.1	458.8	0.812619	0.49607	2.192857
273.8	9.3	25.8	182.3	282.2	303.1	0.665814	3.034409	1.82093

Adult

Resting time			Walked distance			Walking velocity		
Control	LD50	LD90	Control	LD50	LD90	Control	LD50	LD90
231	248.4	105.8	330	379.5	554.4	1.428571	1.527778	2.328922
258	202.8	125.5	243	360.1	566.2	0.94186	1.775641	2.005135
246.7	105.6	174.8	301.2	385.9	511.2	1.220916	3.654356	1.299771
265.4	244.9	101.9	252.1	352.4	574.4	0.949887	1.438955	2.505288
245.5	271.9	128.3	300.8	317.4	500.9	1.225255	1.167341	1.735169
228.4	170.6	137.5	315	336	587.1	1.379159	1.969519	1.897697
263	243	145.1	275.2	389.9	524	1.046388	1.604527	1.605023
277.3	182.2	65.1	291.7	382.3	546.5	1.051929	2.098244	3.731012
216	142.5	72.6	335	348.4	501.2	1.550926	2.444912	3.068258
240.1	213.2	135.7	269.3	325.4	562.2	1.121616	1.526266	1.841317
212.1	336.6	192.5	339.1	316.1	528.1	1.598774	0.939097	1.219278
208.4	138.3	158.4	286.4	385.9	559.9	1.37428	2.790311	1.570988

233.8	205.1	67.5	261.7	351.7	563.4	1.119333	1.714773	3.70963
263.6	172	86.4	265	312.2	575.6	1.005311	1.815116	2.960905
256.2	157.2	190.6	259.7	336	581	1.013661	2.137405	1.354786
200.3	160.1	168.3	233.7	389.9	506.5	1.16675	2.435353	1.337559
